# Assessing Genetic Risk for Physical Activity and Its Interaction with Diet in Predicting Activity Levels and Weight Loss in the iMPROVE Study

**DOI:** 10.3390/genes17020155

**Published:** 2026-01-29

**Authors:** Maria Kafyra, Panagiotis Symianakis, Ioanna Panagiota Kalafati, Panagiotis Moulos, George V. Dedoussis

**Affiliations:** 1 Department of Nutrition and Dietetics, School of Health Science and Education, Harokopio University of Athens, 17676 Athens, Greece; 2 Institute for Fundamental Biomedical Research, Biomedical Sciences Research Center ‘Alexander Fleming’, 16672 Vari, Greece

**Keywords:** obesity, physical activity, polygenic risk score, dietary intervention, gene-diet interactions

## Abstract

Background: Physical activity (PA) and weight regulation are influenced by both genetic and lifestyle factors. This study aimed to evaluate the predictive value of Polygenic Risk Scores (PRSs) for PA and weight outcomes, and their interaction with dietary habits. Methods: Baseline phenotypic data from 202 participants enrolled in the iMPROVE study were analyzed. The sample included 59 men and 143 women, aged 19–65 years. Based on baseline Body Mass Index (BMI), 75 participants were classified as having overweight and 126 as having obesity. Polygenic risk scores (PRSs) were calculated for 197 participants with available genetic data. PA was operationalized as metabolic equivalent of task minutes per week (MET-mins/week), derived from self-reported activity questionnaires. Weight-related outcomes included log-transformed weight loss from baseline to month 3 and change in BMI post-intervention. Interactions with diet were examined using both the randomized intervention dietary groups and previously extracted dietary patterns from the iMPROVE cohort. Correlation analyses and linear regression models were used to assess the main effects of PRSs and dietary patterns, as well as gene–diet interactions. Results: The measured PA PGS002254 presented a nominal significant interaction with diet group for weight loss post-intervention (B = 7.57, SE = 3.57 × 10^0^, *p* = 0.04; R^2^ = 0.06). Similarly, the sedentary behavior PGS001923 presented a significant interaction with the “High in unsaturated fats and fruit juice consumption” pattern for baseline MET-mins/week (B = 1.51 × 10^3^, SE = 4.135 × 10^2^, *p* = 0.001; R^2^ = 0.091). Conclusions: Genetic predisposition influences short-term activity and weight outcomes, with dietary patterns moderating these effects. However, the multifactorial nature of lifestyle behaviors is being underscored by the modest variance explained.

## 1. Introduction

Obesity is identified as a multifactorial disease deriving from the interplay between genetic predisposition and environmental exposures. Heritability estimates from twin and family studies suggest that 40–75% of interindividual variation in body mass index (BMI) can be explained by genetic factors [1,2]. However, lifestyle risk factors, such as dietary and physical activity (PA) habits, remain central determinants of weight regulation and health outcomes. While PA is typically regarded as a modifiable behavior that can counterbalance obesity risk, growing evidence demonstrates that it itself is also partly influenced by increased genetic predisposition, raising important questions about its role as both an outcome of genetic liability and a modifier of obesity susceptibility [3,4].

Heritability estimates for PA behaviors, such as leisure-time PA, moderate-to-vigorous physical activity (MVPA), and daily step count, range between 20% and 60%, highlighting a substantial contribution of genetic influences to their variability [3,4]. Recent genome-wide association studies (GWAS) have identified loci associated with PA, implicating pathways related to motivation, energy balance, and even neurobehavioral regulation [5,6]. Single-nucleotide polymorphisms (SNPs) within MC4R, TMEM18, and SH2B1, long recognized for their role in obesity susceptibility, are also associated with lower PA levels and greater sedentary behavior, suggesting pleiotropic effects that act through both metabolic and behavioral pathways [7,8,9,10]. The consequences of these genetic influences on PA extend directly to weight regulation. Reduced PA lowers energy expenditure and predisposes to positive energy balance, but it may also attenuate responsiveness to behavioral interventions targeting an increase in PA habits [11]. The current literature supports this interpretation, seeing as individuals with higher genetic risk for obesity not only engage in less spontaneous PA but also achieve smaller reductions in body weight and fat mass in response to structured exercise programs, despite demonstrating consistent adherence [12,13,14].

Recent studies suggest that physical activity levels are influenced by both genetic and environmental factors, with dietary habits playing a key role in modulating genetic risk and its subsequent effect on weight regulation. Evidence from behavioral genetics indicates that people genetically inclined toward lower levels of PA may not always exhibit this tendency, as environmental factors, such as dietary habits, can modify how and to what extent such genetic risks are expressed [15]. The latter can be mitigated by dietary habits via mechanisms of epigenetic regulation and metabolism. For example, dietary compounds such as folate, complex B vitamins, and polyphenols are important for regulating both DNA methylation and histone acetylation, which, in turn, regulate the expression of genes related to energy metabolism and neurobehavioral characteristics needed for PA [16]. In 2024, Zhang et al. noted that maintaining a healthy lifestyle, including adhering to a balanced diet, significantly reduced the genetic risk for decreased PA, suggesting that dietary habits can offset genetic susceptibility [17].

Simultaneously, sedentariness, which is often viewed as the behavioral equivalent of low levels of PA, has also demonstrated a heritable component whereby genetics can account for up to 30% of its variance [18]. Anti-inflammatory and nutrient-rich diets may help mitigate the genetic risk for sedentariness by modulating inflammation, energy metabolism, and neurobehavioral pathways [19]. For instance, Wang et al. (2025) found that people adhering to diets of low dietary inflammatory index (DII) values had a significantly lower risk of all-cause mortality despite high levels of sedentary behavior, indicating that diet can buffer the physiological consequences of inactivity [19]. Furthermore, epigenetic mechanisms may also contribute to this interaction. Nutrients, such as polyphenols, omega-3 fatty acids, and B vitamins, can alter gene expression related to the genetic predisposition toward motivation, fatigue, and energy regulation [20,21]. Although evidence directly linking diet to the attenuation of genetic risk for sedentariness has yet to be fully elucidated, these findings provide supportive information to the hypothesis that diet may attenuate the penetrance of risk to inactivity or sedentary behaviors, especially when paired with behavioral strategies. Such associations have also been displayed between dietary patterns and sedentary behavior amongst children and adolescents. In 2025, Trigueros and Aguilar-Parra (2025) emphasized that poor dietary habits during developmental stages can reinforce sedentary tendencies, whereas healthier diets may promote more active lifestyles through improved mood and energy levels [22]. These findings are further supported by evidence that inflammation-related variants have been previously associated with dietary patterns and sedentary behavior in adolescents. This suggests a potential gene–diet interaction whereby pro-inflammatory diets may exacerbate genetic predisposition toward inactivity during critical developmental periods [23].

Despite recent advances, whether genetic predisposition to PA itself influences weight loss outcomes under structured dietary interventions has yet to be fully elucidated. This distinction is critical, as individuals genetically predisposed to lower PA habits may experience greater difficulty in not only maintaining active lifestyles but also achieving weight loss, in the presence of adhering to hypocaloric dietary regimens. Addressing this gap is essential to advancing precision lifestyle interventions that integrate both behavioral and genetic dimensions of obesity risk.

In order to investigate these dynamics, we used data from the iMPROVE study, conducted in Greek adults with overweight or obesity [12,13,14]. Its initial phase assessed the baseline dietary and lifestyle components, capturing diet and PA behaviors [12], while subsequent findings demonstrated that dietary intervention led to significant weight loss and quality of life changes, irrespective of macronutrient composition [13,14]. Building on this foundation, the present study examines whether genetic predisposition for PA predicts activity levels and weight loss trajectories in response to a 6-month dietary intervention, and whether diet interacts with genetic risk for PA to shape these outcomes. By disentangling the dual role of PA—as both a genetically influenced phenotype and a behavioral modifier of genetic obesity risk—this work aims to clarify the pathways through which genes and lifestyle converge to influence weight regulation. Ultimately, it seeks to inform the design of precision-guided interventions, where diet and lifestyle strategies are tailored not only to metabolic profiles but also to genetic liability for PA behaviors.

## 2. Materials and Methods

### 2.1. Study Design and Participants

The iMPROVE study was a dietary intervention conducted to examine the effects of adhering to hypocaloric diets of different macronutrient composition on weight loss in adults with overweight or obesity. The study protocol was approved by the Institutional Review Board at Harokopio University of Athens, and all participants provided written informed consent prior to participation. This study was conducted according to the guidelines of the Declaration of Helsinki and approved by the Research Ethics Committee of Harokopio University of Athens (protocol number 1800/13-06-2019, approval date: 13 June 2019).

The details of the study have been previously described elsewhere [12,13,14]. Participants were eligible if they were aged 18–65 years, had a BMI ≥ 25 kg/m^2^, and were generally healthy and weight-stable for at least 3 months prior to enrollment. Exclusion criteria included pregnancy or lactation at the time of enrolling in the study, diagnosis of diabetes or other chronic metabolic disease affecting weight or appetite, use of medications affecting weight or appetite, and current participation in a structured weight-loss program.

Participants were randomly assigned to one of two hypocaloric diet groups: a high-carbohydrate (55–60% of total energy from carbohydrates, 15–20% from protein, and 20–25% from fat) or a high-protein diet (40% of total energy from carbohydrates, 25–30% from protein, and 30–35% from fat). Both diets were energy-restricted (~500–750 kcal/day below estimated energy needs) based on the resting energy expenditure and baseline activity levels of each individual. Meal plans, dietary guidance, and behavioral support were provided at baseline and reinforced through bi-weekly check-ins via telephone. Participants were advised to maintain their PA at their usual levels throughout the duration of their participation in the intervention.

The primary outcome was a change in body weight and BMI from baseline to the end of the 3-month period. Secondary outcomes included changes in body composition, biochemical indices, and other lifestyles characteristics (sleep quality and overall quality of life). Baseline data were included for participants with available measurements. Body mass index (BMI) and weight were collected for 202 participants. Information on physical activity, expressed as MET-minutes per week, was available for 181 participants. Participants were allocated to the dietary intervention groups as follows: 86 to the high-carbohydrate group and 95 to the high-protein group.

### 2.2. Assessment of Dietary Intake and PA Habits

In addition to allocating each participant to macronutrient-defined groups for the purposes of the intervention, baseline dietary intake data were recorded via a self-reported Food Frequency Questionnaire (FFQ) [24] and were used to derive dietary patterns using principal component analysis (PCA). As previously described, five major dietary patterns were extracted, namely: the Mixed; Med-proxy; Eating-out; Traditional, vegetarian-like; and the High in unsaturated fats and fruit juice consumption [12].

PA was measured at baseline and at the end of each intervention month (months 1–3) using the short form of the International Physical Activity Questionnaire (IPAQ-SF) [25]. Responses were used to derive a physical activity level (PAL) score, which was categorized according to established IPAQ thresholds, namely, (i) sedentary (PAL = 1): <600 metabolic equivalent minutes per week (MET-mins/week); (ii) moderate (PAL = 2): 600–2999 MET-mins/week; and (iii) vigorous (PAL = 3): ≥3000 MET-mins/week. In addition to categorical PAL classification, continuous PA was operationalized as total MET-mins/week, calculated at each time point (baseline, end of month 1, end of month 2, and end of month 3).

### 2.3. Genetic Data and Polygenic Risk Scores (PRSs)

To investigate the impact of genetic predisposition on MET-minutes/week, we calculated already published PA-related Polygenic Risk Scores (PRSs), available in the PGS Catalog. We used genetic data for the iMPROVE study, and we have previously described the procedures of genotyping [13] and further imputed using the European Reference panel of the 1000 genomes Phase 3 panel via use of the plink (version 1.9; Broad Institute, Cambridge, MA, USA) [26] and IMPUTE (version 2; University of Oxford, Oxford, UK) [27] software packages. To ensure maximum coverage of the SNPs included in the candidate PRSs, we performed additional imputation was performed using the Michigan Imputation Server (University of Michigan, Ann Arbor, MI, USA) with the Haplotype Reference Consortium (HRC) reference panel (version r1.1) [28,29].

Three polygenic risk scores (PRSs) were calculated from previously published GWAS. The first was a PRS for measured PA, constructed from accelerometer-derived activity phenotypes (PGS002255) [using accelerometer data for daily moderate-to-vigorous physical activity (MVPA) and daily step count] [30]. The second was a PRS for self-reported PA, developed from leisure-time PA (MET score) derived from self-reporting in the UK Biobank. (PGS002254) [31]. The third was a PRS for sedentary behavior, based on genome-wide associations with device-measured sedentary time (i.e., time spent on watching television or using a computer) (PGS001923) [32]. These scores were selected to capture both objective and subjective dimensions of activity, as well as sedentary lifestyle patterns, enabling a comprehensive evaluation of genetic influences on physical activity and weight-related outcomes.

For each panel, we assessed the following: (i) variant coverage, defined as the proportion of SNPs from each of the selected PGS Catalog scores that were present and usable in our cohort after imputation, and (ii) predictive performance, as measured by the proportion of phenotypic variance explained (R^2^) in our cohort for each PRS, using linear regression models. This dual-panel approach allows for the comparison of both the technical feasibility (coverage) and biological relevance (predictive power) of PRSs derived from different imputation sources, thereby informing best practices for PRS implementation in diverse cohorts.

### 2.4. Statistical Analysis

For the present analyses, baseline measurements were obtained during the in-person assessment, while participant-reported data collected at the end of each subsequent month (up to three months) were used to evaluate changes from baseline. Due to the non-normal distribution of variables, descriptive statistics were reported as median and interquartile range (IQR). Baseline characteristics were compared between sexes and diet groups using the Mann–Whitney U test for independent samples and the Wilcoxon signed-rank test for paired comparisons, as appropriate. Analyses were conducted on a per-protocol basis, including only participants who completed the 3-month intervention. No imputation of the missing PA data was performed; participants who discontinued the study or had incomplete follow-up data were excluded from the analysis.

To assess associations between genetic predisposition and PA, Pearson correlation coefficients were computed between each PRS and PA measures at baseline and three follow-up points (months 1–3). Furthermore, PRS low vs. high risk groups were created via dichotomization of the PRSs based on the sample’s reported median values (attribution of a value of 1 for scores below the sample’s median and a value of 2 for scores above the observed median). To evaluate the predictive performance of each of the PRSs based on the two imputation panels, we proceeded with fitting regression models with and without each PRS, adjusting for relevant covariates [i.e., age, sex, smoking (1 = Non-smoker and 2 = Smoker), and diet group]. The incremental r squared (R^2^) was calculated as the difference in explained variance between the full model (including the PRS) and the null model (covariates only).

To evaluate the effects of diet, PA PRSs, and their interactions on weight loss, linear regression models were fitted with weight change as the dependent variable. Independent variables included diet group (high carbohydrate vs. high protein), each of the respective PRSs, and their interaction term (diet × PA PRS), as appropriate. Linear regression models were adjusted for age, sex, and smoking status. Additionally, in order to further investigate the potential interactions with baseline dietary habits, we further examined associations between the interactions of said PRSs and the previously calculated dietary patterns. Variables not following the normal distribution were log-transformed when used as dependent variables. Model variance explained was estimated via R^2^.

All analyses were performed using R and Plink version 1.9, and significance was set at α = 0.05 (interaction analyses of the five dietary patterns and the six PRSs included an adjusted statistically significance threshold of α = 0.05/30 = 0.002).

## 3. Results

### 3.1. Descriptive Statistics

Overall, we used available baseline data for 202 participants for BMI and weight data and 181 participants for information on MET-mins/week (Table 1). Of the 181 participants enrolled at baseline, only 79 completed the 3-month intervention, reflecting a substantial dropout rate. We used the baseline vs. end-of-month 3 data to investigate the causes for dropout. The only significant baseline difference between participants who completed the 3-month study and those who dropped out was age, with dropout occurring more frequently among younger participants (*p* = 0.002), potentially suggesting a greater interest in well-being by participants who were older in age. The only significant difference observed between participants who completed the study at 3 months and those who dropped out was in age, suggesting dropout was more frequent in younger participants (*p* = 0.002). PA level (as shown in MET-min/week) differences between males and females were not statistically significant at any time point (*p* > 0.05). In contrast, weight and BMI demonstrated consistent reductions over time, with females showing significantly lower values than males at all measured intervals (*p* < 0.001 for weight; BMI differences were not statistically significant).

More specifically, PA as assessed by IPAQ-derived MET-mins/week, did not present statistically significant differentiations across the three time-points (Table 1), as instructed at baseline to the participants not to alter usual PA levels. Only participants in the high protein group demonstrated a slight decrease in MET-mins/week from baseline to the end of month 1 (*p* = 0.046). Within-diet group differences across the 3 months also presented no statistically significant changes. Regarding changes in BMI or weight change between groups, there were no significant differences, although both groups showed significant reductions in BMI and weight (*p* < 0.001 for all).

### 3.2. Predictive Utility of PRS for PA and Weight-Related Outcomes

Regarding the impact of imputation reference panels on PRS coverage, using the 1000 genomes phase 3 imputation panel, coverage for PGS002255 was 570,759 out of 1,140,081 SNPs (50%); for PGS002254, it was 571,001 out of 1,142,321 SNPs (49.9%); for PGS001923, it was 26,650 out of 50,583 SNPs (53%). In contrast, imputation with the HRC panel yielded higher coverage across all scores: 807,657 SNPs for PGS002255 (71%), 808,197 SNPs for PGS002254 (71%), and 36,787 SNPs for PGS001923 (73%).

To assess the predictive utility of PRS for baseline PA using MET-mins/week, we compared two linear regression models: a null model including standard covariates (age, sex, smoking status, and diet group), and a full model that additionally incorporated each PRS. Using genotype data imputed with the 1000 genomes phase 3 panel, the inclusion of PGS002255 increased the explained variance in baseline MET-mins/week from 0.9% (R^2^ = 0.009) to 1.76% (R^2^ = 0.0176), yielding an incremental R^2^ of 0.009. Similarly, the addition of PGS002254 and PGS001923 resulted in incremental R^2^ values of 0.008 and 0.011, respectively.

When using genotype data imputed with the HRC panel, predictive performance improved across all scores. The full model, including PGS002255, explained 2.2% of the variance (R^2^ = 0.022), corresponding to an incremental R^2^ of 0.013. Comparable gains were observed for PGS002254 (incremental R^2^ = 0.013) and PGS001923 (incremental R^2^ = 0.027), indicating that imputation with the HRC panel consistently enhanced the predictive power of PRSs for baseline PA levels. However, none of the PRSs were statistically significant predictors in the full models, and their overall model fits did not reach statistical significance.

**Figure 1:**
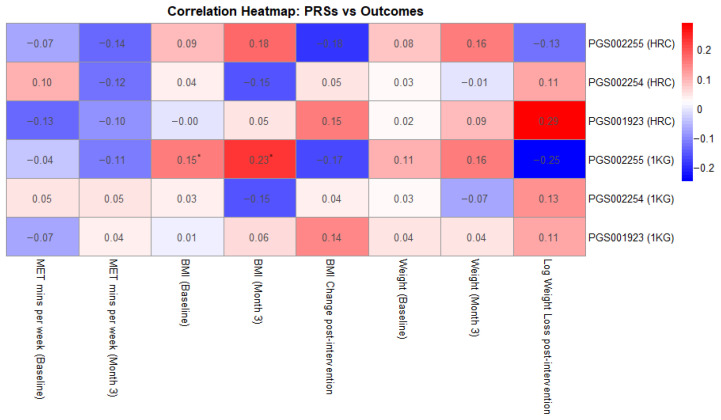
Correlation heatmap between the examined PRSs and key intervention measurements (* showing statistically significant results).

Additionally, as shown in Figure 1, we investigated correlations between the examined PRSs and MET-mins/week as well as weight loss across the 3-month intervention period. None of the PRSs showed statistically significant correlations with MET-minutes/week, either at baseline or post-intervention, with correlation coefficients ranging from −0.13 to 0.10, *p*-values exceeding 0.08, and confidence intervals crossing zero. Similarly, no statistically significant associations were observed between PRS and BMI change or weight loss, with coefficients ranging from −0.18 to 0.19. The strongest trend was observed for PGS001923 (HRC-imputed), which showed a weak positive correlation with weight loss (r = 0.187, *p* = 0.093), suggesting a potential association that warrants further investigation.

The only statistically significant associations were observed for PGS002255 imputed with the 1000 Genomes panel, which showed a modest, positive correlation with baseline BMI (r = 0.154, *p* = 0.031) and with BMI at month 3 (r = 0.232, *p* = 0.036). Associations with baseline and post-intervention body weight were similarly weak and non-significant, with correlation coefficients ranging from 0.02 to 0.16 and *p*-values above 0.14.

### 3.3. Differences in Weight Loss per PRS Group

Participants with higher genetic predisposition for PA showed significant reductions in weight from baseline to month 3 across multiple PGS groups (e.g., PGS002255: Z = −5.022, *p* < 0.001; PGS002254: Z = −4.320, *p* < 0.001; PGS001923: Z = −4.015, *p* < 0.001), whereas participants with lower genetic predisposition did not demonstrate significant weight change (*p* = 0.655). These findings suggest that genetic predisposition for increased PA modulated the response to intervention with respect to weight change, while PA levels remained largely unchanged within diet groups (changes in PA are summarized in Appendix A).

### 3.4. PRS-Diet Effects on PA and Weight Change

Regarding PA outcomes (MET-mins/week), PGS002255 (HRC panel) showed a nominally significant negative association at the end of month 1 (Β = −3.73 × 10^7^, *p* = 0.021) (Table 2), indicating that individuals with higher genetic risk scores were less likely to increase their activity levels during the early phase of the intervention. No significant main effect was observed for BMI change post-intervention (*p* = 0.174).

**Figure 2:**
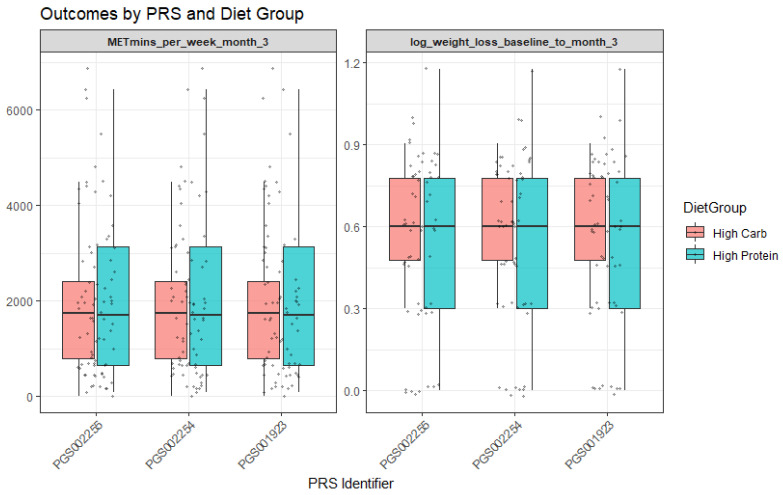
Outcomes by PRS and diet group.

For PGS002254, baseline analyses revealed interactions with the diet group. More specifically, participants assigned to the high-protein diet group demonstrated greater log weight loss (Β = −1.17, *p* = 0.02). Moreover, the PGS002254 × diet group interaction was nominally significant (Β = 7.57, *p* = 0.04; R^2^ = 0.06), suggesting that genetic predisposition moderated the effect of dietary assignment on weight loss. In practical terms, individuals with higher PGS002254 values appeared to benefit more when allocated to the high-protein group (Figure 2). Finally, for PGS001923, a significant moderation effect was observed with the “High in unsaturated fat and fruit juice consumption” dietary pattern (interaction Β = 1.51 × 103, *p* = 0.001; R^2^ = 0.091). This indicates that adherence to this dietary pattern amplified the genetic influence on outcomes, with participants carrying higher genetic risk showing distinct responses when adhering to the dietary pattern rich in unsaturated fats and fruit juice.

## 4. Discussion

Using data from the iMPROVE three-month weight-loss dietary intervention, we sought to determine whether genetic predisposition for PA and sedentary behavior modifies weight loss under different macronutrient dietary regimens (high-carbohydrate vs. high-protein). Our results show that while diet remained the dominant predictor of weight loss, there were some modest gene–diet interactions, involving PRSs for PA behaviors (such as measured and self-reporting PA and time spent watching TV or on the computer) under the adherence to specific dietary patterns, although the genetic effects were small and explained little variance.

Although imputation with the HRC panel yielded higher SNP coverage across all PRSs, this did not translate into statistically significant predictive power when the scores were included in regression models alongside key covariates (age, sex, smoking status, and diet group). This outcome highlights an important nuance, referring to the fact that while higher coverage generally improves the fidelity of PRS construction, it does not guarantee stronger associations with complex traits like PA or weight loss [33]. The lack of statistical significance may reflect the modest effect sizes of individual SNPs, the multifactorial nature of the outcomes, or residual confounding [34].

Additionally, statistical significance in correlation does not always translate to meaningful variance explained in regression models, especially when effect sizes are small and confounding factors are present [35,36]. These findings underscore the importance of evaluating PRS performance not only by technical metrics like coverage, but also by their incremental contribution to explained variance and statistical robustness within multivariable models. Additionally, they underscore the complexity of PRS performance, where imputation quality, variant informativeness, and trait architecture all interact, as previously shown in PRS development using cohorts of various sizes [37,38].

The measured PA for PGS002255 showed gene–diet interaction signals with diet group at the end of month 1, suggesting that genetic predisposition for PA might be modulated by the dietary context, specifically in the allocation to the high-protein group. The self-reported PA for PGS002254 showed a significant interaction with diet group, highlighting that individuals with increased genetic risk for PA can benefit from adhering to a high-protein diet to achieve better weight loss. Mechanistically, diets high in protein could help compensate for genetic predispositions to low PA by increasing thermogenesis, satiety, and preserving lean body mass [39].

Importantly, sedentary behavior was analyzed as a distinct construct from PA, consistent with current consensus in the literature. The sedentary behavior for PRS001923 was based on hours spent watching television or using the computer, and it demonstrated an interaction with the “High in unsaturated fat and fruit juice consumption” dietary pattern in increasing baseline MET-mins/week. This distinction is critical, as individuals may simultaneously meet PA guidelines while engaging in prolonged sedentary time, and our findings reflect genetic predisposition specifically for sedentary time rather than a general “sedentary vs. active” lifestyle. Likewise, dietary patterns rich in unsaturated fats, fruit, and adhering to Mediterranean-type features may provide metabolic flexibility, reduce inflammation, or otherwise ameliorate the negative effects of sedentariness. These mechanisms are consistent with those proposed in the obesity genetics × diet quality literature [39].

Our results suggest that genetic risk for PA and sedentary behavior may more strongly interact with certain dietary patterns, namely those higher in protein or richer in unsaturated fats and fruit juice. This finding is in line with observational meta-analytic evidence showing that higher diet quality attenuates the effect of obesity polygenic risk on BMI and waist circumference [40]. Overall, dietary habits assessed via adherence to extracted dietary patterns have been previously shown to mediate or attenuate genetic predisposition for not only anthropometric but also inflammatory indices [23,41,42]. While many studies on diet and PRS interactions focus on obesity risk rather than weight loss trials per se, the current literature provides limited evidence for the effects of genetic background on PA as a lifestyle factor. For example, a systematic review and meta-analysis found that individuals with higher adherence to plant-based or high-quality dietary patterns had weaker associations between genetic risk and obesity-related anthropometric outcomes [42,43].

Although interaction signals were present, direct genetic effects masked very little weight loss, with diet group assignment remaining the most important predictor for weight change. This mirrors findings from trials in which macronutrient differences drive weight/fat mass loss more strongly than other modifiers when energy intake is controlled [43].

Despite the consistent-with-literature demonstrated findings, our study has several limitations: (i) the 3-month duration may be insufficient to detect longer-term gene × diet interactions or to assess sustainability of weight loss; (ii) our sample size, while usually adequate, may still limit power for detecting small genetic effects, especially interactions; (iii) the use of self-reporting for some PA metrics could introduce measurement error, weakening associations. Additionally, dietary-pattern factors are derived post hoc via factor analysis and may not align perfectly with standardized definitions of Mediterranean diet or unsaturated fat-rich diets in other studies.

## 5. Conclusions

In summary, our results suggest that while genetic predisposition for sedentary behavior or low physical activity does modestly interact with dietary patterns to influence weight loss, these interactions are small, and diet composition—especially higher protein intake and higher quality dietary patterns—is the more potent determinant. These findings support the potential value of personalizing weight loss interventions by dietary quality in addition to macronutrient distribution, especially for individuals with higher genetic risk for sedentariness. Future, longer, larger studies with richer phenotyping of behavior and activity are needed to clarify who benefits most from which diet in the context of genetic risk.

## Figures and Tables

**Table 1:** Changes in MET-minutes, weight, and BMI during the three months of the intervention, per diet group (adapted by [12]).

Variable	Time	iMPROVE Cohort	Median (IQR)	High-Carb Group	Median (IQR)	High-Protein Group	Median (IQR)	*p* *
PA (MET-mins/week)	Baseline	181	1280 (1623)	86	1285 (1545)	95	1280 (1683)	0.911
	Month 1	126	1100.5 (1524)	58	1224 (1762)	68	1041 (1491)	0.224
	*p* **	0.336			0.488		0.046	
	Month 2	91	1187 (1904)	42	1445 (1873)	49	945 (2261)	0.325
	*p* **	0.564			0.550		0.861	
	Month 3	79	1638 (2175)	36	1737 (1817)	43	1638 (2518)	0.910
	*p* **	0.411			0.854		0.188	
	*p* ***	0.271			0.701		0.264	
Weight (kg)	Baseline	202	87 (26)	94	83.50 (26)	108	88.50 (25)	0.014
	Month 1	118	84 (25)	56	81.50 (21)	62	86 (26)	0.173
	*p* **	<0.001			<0.001		<0.001	
	Month 2	89	82 (25)	42	80 (20)	47	86 (25)	0.149
	*p* **	<0.001			0.047		0.006	
	Month 3	84	83 (25)	36	79 (25)	48	84.5 (20.5)	0.178
	*p* **	0.819			0.478		0.843	
	*p* ***	<0.001			<0.001		<0.001	
BMI (kg/m^2^)	Baseline	202	31.35 (6.9)	94	30.5 (6.9)	108	32.3 (7.8)	0.920
	Month 1	118	30.14 (6.08)	56	29.58 (6.34)	62	31.18 (6.09)	0.249
	*p* **	<0.001			<0.001		<0.001	
	Month 2	89	29.71 (6.12)	42	29.84 (5.43)	47	29.71 (7.02)	0.421
	*p* **	<0.001			0.088		0.006	
	Month 3	84	29.43 (6.50)	36	29.21 (6.83)	48	29.47 (6.32)	0.333
	*p* **	0.867			0.458		0.610	
	*p* ***	<0.001			<0.001		<0.001	

*: *p*-value showing differences within the two diet groups, using the Mann–Whitney test. **: *p*-value showing change from the previous month for each diet group, using the Wilcoxon signed-rank test. ***: *p*-value showing overall change from baseline using the Wilcoxon signed-rank test.

**Table 2:** Associations of PRSs with PA (MET-mins/week) and weight loss post-intervention.

PRS	Outcome Type	Imputation Panel	Timepoint	Significant Interactions	Model Fit (R^2^)
PGS002255	MET-mins/week	HRC	Month 1	PGS002255, Β = −3.729 × 10^7^, SE = 1.59 × 10^4^, *p* = 0.021 DietGroup, Β = 1.531 × 10^3^, SE = 1.011 × 10^3^, *p* = 0.13 PGS002255 × DietGroup, Β = 2.125 × 10^7^, SE = 9.84 × 10^3^, *p* = 0.059	0.092
PGS002254	Log Weight Loss post-intervention	HRC	Post-intervention	PGS002254 Β = −1.083 × 10, SE = 6.11 × 10^0^, *p* = 0.08 Diet Group, Β = −1.172 × 10^0^, SE = 5.063 × 10^−1^, *p* = 0.02 PGS002254 × Diet Group, Β = 7.572 × 10^0^, SE = 3.57 × 10^0^, *p* = 0.04	0.06
PGS001923	MET-mins/week	HRC	Baseline	PGS001923, Β = −8.460 × 10^2^, SE = 4.25 × 10^2^, *p* = 0.07 High unsaturated fats and fruit juice consumption, Β = −8.428 × 10^2^, SE = 2.932 × 10^2^, *p* = 0.005 PGS001923 × High unsaturated fats and fruit juice, Β = 1.511 × 10^3^, SE = 4.135 × 10^2^, *p* = 0.001;	0.091

## Data Availability

The data presented in this study are available on request from the corresponding author. The data are not publicly available due to participants’ privacy and ethical restrictions.

## Appendix A. Differences in PA per Diet Group

Changes in physical activity, measured as MET-minutes per week, were examined across the three time points within each diet group using the Wilcoxon signed-rank test. Specifically, the Wilcoxon signed-rank tests showed that differences between month 1 and month 2 (Diet Group 1: Z = −0.598, *p* = 0.550; Diet Group 2: Z = −0.175, *p* = 0.861), month 2 and month 3 (Diet Group 1: Z = −0.184, *p* = 0.854; Diet Group 2: Z = −1.316, *p* = 0.188), and baseline and month 3 (Diet Group 1: Z = −0.384, *p* = 0.701; Diet Group 2: Z = −1.116, *p* = 0.264) were not statistically significant.
